# Tungsten-Doped Zinc Oxide and Indium–Zinc Oxide Films as High-Performance Electron-Transport Layers in N–I–P Perovskite Solar Cells

**DOI:** 10.3390/polym12040737

**Published:** 2020-03-26

**Authors:** Ju Hwan Kang, Aeran Song, Yu Jung Park, Jung Hwa Seo, Bright Walker, Kwun-Bum Chung

**Affiliations:** 1 Department of Materials Physics, Dong-A University, Busan 49315, Korea; kangjuhwan89@gmail.com (J.H.K.); j6768kr@gmail.com (Y.J.P.); 2 Division of Physics and Semiconductor Science, Dongguk University, Seoul 04620, Korea; aeransong@dongguk.edu; 3 Department of Chemistry, Kyung Hee University, Seoul 02447, Korea

**Keywords:** transparent metal oxide, perovskite solar cell, tungsten-doped InZnO, zinc–oxynitride

## Abstract

Perovskite solar cells (PSCs) have attracted tremendous research attention due to their potential as a next-generation photovoltaic cell. Transition metal oxides in N–I–P structures have been widely used as electron-transporting materials but the need for a high-temperature sintering step is incompatible with flexible substrate materials and perovskite materials which cannot withstand elevated temperatures. In this work, novel metal oxides prepared by sputtering deposition were investigated as electron-transport layers in planar PSCs with the N–I–P structure. The incorporation of tungsten in the oxide layer led to a power conversion efficiency (PCE) increase from 8.23% to 16.05% due to the enhanced electron transfer and reduced back-recombination. Scanning electron microscope (SEM) images reveal that relatively large grain sizes in the perovskite phase with small grain boundaries were formed when the perovskite was deposited on tungsten-doped films. This study demonstrates that novel metal oxides can be used as in perovskite devices as electron transfer layers to improve the efficiency.

## 1. Introduction

Organic–inorganic perovskite solar cells (PSCs) are viewed as promising next-generation optoelectronic devices due to their superior optical and electrical properties, simple solution handling and low cost [1,2,3,4,5]. In particular, the power conversion efficiency (PCE) of PSCs has increased to more than 20% over the past decade [6,7]. Organic–inorganic hybrid lead halide perovskites possess excellent properties for solar cell applications, such as strong light harvesting ability (absorption coefficients, α, of more 5 × 10^4^ cm^-1^), high carrier mobility (0.1–10 cm^2^·V^-1^∙s^-1^) and long diffusion lengths (0.1–1.5 µm) [8,9,10,11]. The PCE of PSCs has improved rapidly due to the development of techniques to control film morphology, effective device architectures, interface engineering, and a constantly growing knowledge base of techniques to control and optimize electronic band structures and semiconducting properties. The most commonly used device architecture comprises a N–I–P structure using a sintered (>450 °C) mesoporous TiO_2_ (mp-TiO_2_) layer as the electron-transport layer (ETL)—yielding PCEs of up to 22.1% [12,13]. However, high-temperature processes are not favorable for device fabrication and hinder the development of flexible modules [14]. To overcome these obstacles, researchers have focused on planar devices using low-temperature solution-treated transport layers. Currently, the most commonly used oxides include TiO_2_ [2,15], ZnO [16] although a variety of other metal oxides [17,18], such as Nb_2_O_5_ [19] and SnO_2_ [20,21], may also be used, allowing some control of the interfacial properties and variability in junctions formed between electrodes and the CH_3_NH_3_PbI_3_ layer. Recently, reports of new metal oxide-based PSCs have demonstrated that PCEs in the range of 15~20% are possible via development and optimization of new oxide layers [22,23,24,25,26,27,28,29,30]. These inorganic oxides are considered excellent interfacial materials due to their superior stability and electrical properties. These interfacial metal oxide layers generally facilitate the extraction of n-type charge carriers from the active layer and act as effective cathode interlayers. Although TiO_2_ and ZnO themselves are fairly well understood [31,32], there are a wide variety of other metal oxides and mixtures which may be used as ETLs in PSCs, which are not well optimized. The composition of metal oxide semiconductors can be used to control the electrical characteristics and additionally, for metal oxide films deposited under a partial vacuum, the oxygen partial pressure in the deposition process can be used to control the composition and properties of films [33,34,35]. Zinc–oxynitride (ZnON) [36,37], Zinc oxide (ZnO) [38,39], Indium–zinc oxide (IZO) [40,41], and W-doped indium–zinc oxide (WIZO) [42,43], for example, are metal oxide semiconductors which have been investigated as channel materials for thin-film field-effect transistors in display backplanes and other optoelectronic devices due to their high transparency, high mobility, and high conductivity. Notably, the element W in WIZO thin films can be used to control the electronic structure, including the band alignment, oxygen-deficient bonding states, and band edge states below the conduction band. In addition, the W doping concentration in WIZO thin films may affect the electronic structure and can be used to control the device performance and stability characteristics [44]. Although W-based oxide films have yielded PCEs of up to 13%, they have received considerably less attention than other oxides such as Zn, Sn, In and Ni [28,41,44]. In this work, we report the fabrication of high-efficiency PSCs based on the architecture: ITO/metal oxide/PC_61_BM/CH_3_NH_3_PbI_3_/Spiro-OMeTAD/MoO_3_/Ag. In this configuration, metal oxides are deposited by sputtering as an ETLs and are additionally modified by including an n-type, organic PC_61_BM layer as an electron extraction layer (EEL) [45,46,47,48,49,50,51,52], which improves wettability of the perovskite solution on the substrate and passivates the oxide surface for perovskite, reducing the density of the trapped states [53,54,55]. The WIZO film exhibited the lowest conduction band and improved open-circuit voltage (V_OC_) compared to other metal oxide films. Moreover, the WIZO film enhanced the crystallinity of the perovskite film and remarkably reduced leakage current in the PSCs. The optimized PSC showed different grain sizes in SEM images compared to other metal oxide films. In addition, the encapsulated PSCs with the WIZO film showed excellent long-term stability under ambient operating conditions. The N–I–P device structures fabricated with metal oxide ETLs produced PCEs of up to 16.44%. To the best of our knowledge, this constitutes the highest performance yet reported for devices based on W oxides and demonstrates the great potential of this new material as ETLs for PSCs and for other potential applications [42,43].

## 2. Experimental Section

### 2.1. Fabrication Process of the ZnON and WIZO Films

Pre-patterned ITO substrates (1.5 × 1.5 cm, 7 Ω/sq) were immersed in deionized water (DI Water), acetone, isopropanol solution (IPA) and ultra-sonication for 20 min in each solvent. The washed ITO substrates were stored in an oven at 80 °C and treated with ozone using a UV-ozone cleaner immediately prior to use. 20 nm thick ZnON films were next deposited by reactive magnetron sputtering using a 3” diameter Zn target with a purity of 99.99% in a reactive gas (Ar/N_2_/O_2_) without substrate heating [56]. The gas delivery system used precision mass flow controllers to control the flow rate of each gas. Sputtering used a direct current (DC) power of 100 W and a working pressure of 5 mTorr. As-deposited ZnON thin films were post-processed by thermal annealing at 300 °C for 1 h in an ambient air. WIZO films (20 nm thick) were deposited onto ITO films via the co-sputtering of a WO_3_ and IZO (1:1 at. %) sputtering target using a radio-frequency (RF) sputtering system without substrate heating [57]. The processes pressure was set at 5 mTorr and the relative oxygen flow rate O_2_/(Ar + O_2_) ratio was 0.05. To change the W doping concentration in the WIZO films, we controlled the deposition rate through the variation of the input RF power of the WO_3_ target from 5 to 20 W while fixing that of the IZO target at 150 W. WIZO thin films were post-processed by thermal annealing at 250 °C for 1 h in an air atmosphere.

### 2.2. Device Fabrication

All samples subsequently were brought into the N_2_ glove-box for the spin coating of PC_61_BM, which was spin-cast at 2000 rpm for 30 s followed by annealing at 80 °C for 10 min (Chemin St-François, Dorval, Canada). A 2.5 wt.% solution of PC_61_BM in chloroform/chlorobenzene (CB) (1:1 volume ratio) was used. 1.1 M solutions of PbI_2_ (99.99%, Sigma Aldrich, Merck KGaA, Darmstadt, Germany) and methylammonium iodide (Sigma Aldrich, 99.5%) were dissolved in a mixed DMF and DMSO solvent (7:3 ratio) with stirring at 60 °C for 60 min. After that, the solution was deposited via a solvent engineering method by spin coating the perovskite precursor solution at 3500 rpm for 30 sec and at 6500 rpm for 5 sec, respectively. Anhydrous chlorobenzene (45 µL) was dripped at the center of the substrate in the second step as the prepared film was put onto the hot plate at 100 °C for 10 min under N_2_ atmosphere. The hole transfer layer was prepared by spin coating a solution consisting of 80 mg of spiro-OMeTAD 8.4 μl of 4-tert-butylpyridine, and 51.6 μl of bis (trifluoromethane) sulfonamide lithium salt (Li-TFSI) solution (154 mg/mL in acetonitrile) all dissolved in 1 mL CB for 30 sec at 4000 rpm. The spiro-OMeTAD layer was aged overnight in the dark under an atmosphere of dry air to promote oxidation and doping. Finally, MoO_3_ (5 nm) and Ag (100 nm) contacts were thermally evaporated onto the spiro-OMeTAD.

### 2.3. Characterization

Current density–voltage (J-V) measurements were collected using a Keithley 2400 source measure unit inside a nitrogen filled glove-box using a high-quality optical fiber to guide the light from a xenon arc lamp to the solar cell devices. The solar cell devices were illuminated with a light intensity of 100 mW/cm^2^ calibrated using a standard silicon reference cell immediately prior to testing. External quantum efficiency (EQE) measurements were carried out using a QEX7 system manufactured by PV Measurements, Inc (Point Roberts, WA, USA). Atomic force microscopy (AFM) images were obtained using a Veeco Multimode microscope operating in tapping mode. X-ray diffraction (XRD) patterns were collected using a Bruker AXS D8 advance diffractometer (Bruker AXS, Karlsruhe, Germany). UV–vis spectra were taken using and Agilent Cary 5000 UV–vis spectrometer. Ultraviolet photoelectron spectroscopy (UPS) spectra were obtained using a Thermo Fischer Scientific ESCALB 250XI (Waltham, MA, USA).

## 3. Results

**Figure 1:**
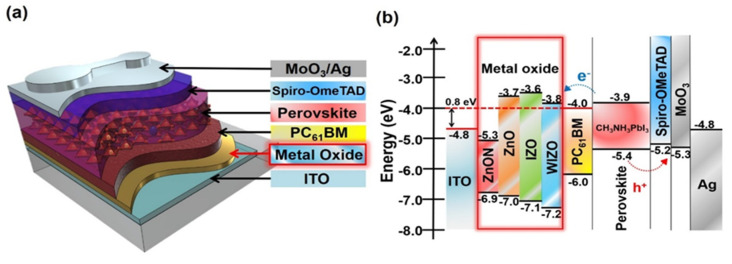
Schematic diagrams. (**a**) Device architecture of N–I–P perovskite cells with various metal oxide layers and (**b**) corresponding energy band diagram (all energies are relative to the vacuum level).

**Figure 2:**
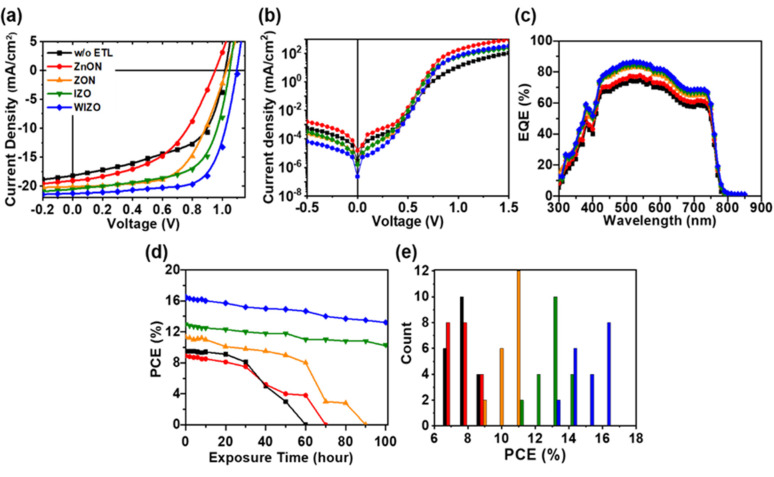
Current Density *vs* Voltage curves measured (**a**) under simulated AM1.5G solar light and (**b**) under dark. (**c**) EQE spectra, (**d**) stability properties after exposure in Air and (**e**) statistics of the performance for all devices corresponding to (**a**).

**Figure 3:**
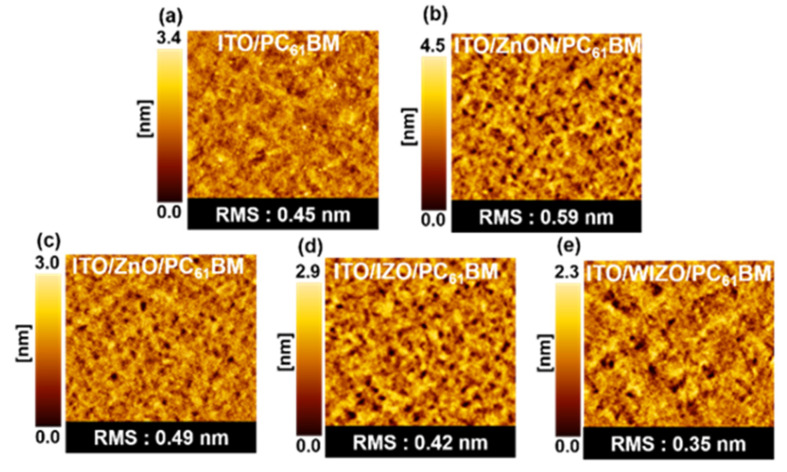
Topographic images (size: 5 μm × 5 μm) of (**a**) ITO/PC_61_BM, (**b**) ITO/ZnO/PC_61_BM, (**c**) ITO/ZnON/PC_61_BM, (**d**) ITO/IZO/PC_61_BM, and (**e**) ITO/WIZO/PC_61_BM films.

**Figure 4:**
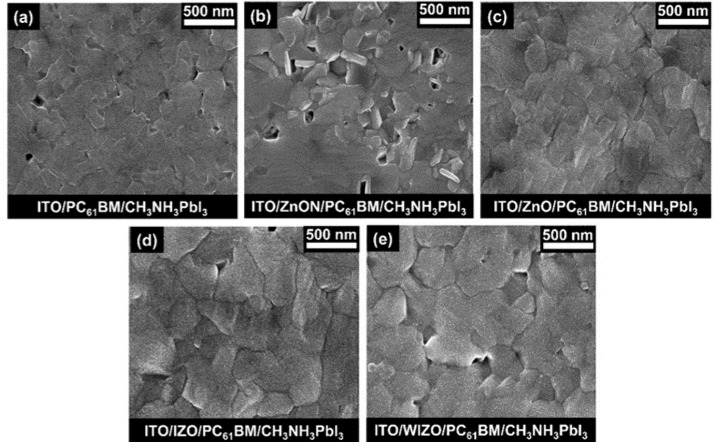
SEM images of CH_3_NH_3_PbI_3_ thin films on PC_61_BM prepared on different metal oxides. (**a**) w/o ETL, (**b**) ZnON, (**c**) ZnO, (**d**) IZO, and (**e**) WIZO.

The device structure used in this study is shown in Figure 1a. In this configuration, a thin metal oxide film was first deposited on ITO glass by sputtering, followed by a light harvesting PC_61_BM/CH_3_NH_3_PbI_3_ junction [58,59], which was deposited by spin coating. Additional details concerning experimental methods are included in the Supplementary Information. Figure 1b shows the energy alignment in the PSCs; in this system photogenerated electrons migrate to the PC_61_BM, and are transported through the metal oxide films before being collected at the ITO cathode. Photogenerated holes migrate to the spiro-OMeTAD/MoO_3_ layers and are collected by the Ag anode [60,61]. The band energies of each material were taken from the literature, except for those of metal oxides. The valence band energies of the metal oxides were obtained by UPS and the conduction band energies were obtained by adding the reported optical bandgap of each material to their valence band energies (Figure S1, Supporting Information) [62]. According to a previously reported transistor, WIZO mobility and energy levels are 19.57 cm^2^/Vs and 3.4 Ev [44]. The Schottky barrier of ITO and PC_61_BM is 0.8 eV when metal oxides are not used. This energetic barrier is expected to limit electron extraction efficiency and promote electron-hole recombination [63]. This indicates that the performance of PSCs may be improved by reducing the recombination of electrons and holes by creating an Ohmic contact using a metal oxide to reduce the energy barrier between the conduction band of the semiconductor and the cathode. In terms of energy levels, WIZO possesses a smaller barrier to electron extraction, indicating that electron extraction should be higher than other metal oxide films by making an Ohmic contact with PC_61_BM. To probe the effect of metal oxides on device performance, PSCs with and without ETLs were fabricated as described in the experimental section. Figure 2a shows the J-V curves for these cells. The corresponding photovoltaic parameters of the PSC performance are given in Table 1. Devices without ETLs yielded a V_OC_ of 1.00 V, a short circuit current density (J_SC_) of 18.20 mA/cm^2^ and a fill factor (FF) of 54.7%, corresponding to a PCE of 9.95%. The V_OC_ was relatively high, but the J_SC_ and FF were very low, which can be attributed to inefficient electron extraction arising from the Schottky barrier at the ITO/PC_61_BM interface. The poor FF in particular leads to a low efficiency. When ZnON was used as an ETL, the devices exhibited an even lower PCE of 8.9% with a V_OC_ of 0.95 V, a J_SC_ of 19.06 mA/cm^2^ and an FF of 48.7. This can be attributed to the large conduction band offset between ZnON and PC_61_BM layer (Figure S1, Supporting Information), together with the low grain size and high surface roughness of ZnON layer (Figure 3 and Figure 4). Therefore, despite having a high electron mobility, the ZnON layer was not able to improve the charge transport, and instead caused increased contact resistance. Replacing ZnON with ZnO led to a significant enhancement in device efficiency. The device with ZnO yielded a PCE of 11.31% with a J_SC_ of 20.09 mA/cm^2^, a V_OC_ of 1.02 V and an FF of 54.8 %. Among the oxides tested, however, devices with IZO and WIZO layers showed the best performance. PSCs based on WIZO yielded a J_SC_ of 21.28 mA/cm^2^, a V_OC_ of 1.10 V, an FF of 70.0% and a PCE of 16.44%. Among the different metal oxides, WIZO yielded the best performance by a significant margin.

IZO devices show a higher PCE than other metal oxide devices; however, they are disadvantageous in electron transport due to the large conduction band offsets which promotes the recombination of electrons and holes (Figure S1). The V_OC_ in a solar cell is governed by many factors; however, the presence of non-radiative recombination pathways for electrons and holes is thought to be one of the primary loss mechanism which reduces the V_OC_ to less than theoretical values in PSCs, and this most likely originates from the presence of defect sites which cause carrier trapping and trap assisted recombination [63]. Additionally, any imperfections in solar cells fabrication which introduce shunting paths will also act to reduce the V_OC_ by increasing the dark leakage current [63,64,65]. The average FFs obtained with four different ETLs are 48.7%, 54.8%, 61.5% and 70.0%, respectively, with the W-doped film showing a significant advantage compared to the other oxide films. The *J*–*V* characteristics of the devices collected in the dark are presented in Figure 2b and the device area-normalized data are plotted on a logarithmic scale. In general, smaller series resistance (R_s_) and larger shunt resistance (R_SH_) will result in larger FF. Here, the increase in FF is due to both a decrease in R_s_ and an increase of R_SH_, which may arise from better contact between perovskite film and metal oxide/PC_61_BM film, higher perovskite film coverage, and better hole-blocking abilities after with ETLs [66,67].

In Figure 2c, the EQE spectra for all devices show similar spectral features, consistent with the identical active layers used in each device. The EQE of the devices without metal oxides and or with ZnON films showed similar spectral profiles due to the low optical density of these materials in the visible spectrum. Although devices without metal oxides have similar optical properties as the metal oxide devices, the relatively low efficiency in this condition is consistent with the lower built-in potential across the perovskite layer and low carrier extraction efficiency. Additionally, metal oxides possess very deep valence band energies which block the back-diffusion of holes, a process which leads to carrier recombination at the cathode in devices without metal oxide interlayers.

Considering the practical application of perovskite solar cells in the future, there is a pressing need for low-cost renewable energy that can potentially be provided by PSCs; however, it is still an enormous challenge to slow down the degradation of sensitive perovskite films under operating conditions [68,69]. To evaluate the effect of the different oxide layers on stability, we monitored bare devices without any encapsulation, and measured PSC characteristics daily over the course of 5 days in ambient air. PCE vs storage time data are shown in Figure 2d. Surprisingly, the PCE of the WIZO-based device remained at 91.4% of its original value after 50 h of storage, and retained over 88.5% of the original PCE even after 100 hours. Likewise, for IZO-based devices, the PCE remained at 84.7% even after 100 h. By contrast, the degradation of ITO-based devices (without ETLs) and ZnON accelerated after 30 h, decreasing to 68.4% PCE after 50 h storage and reaching 0% of the original PCE after only 60 h. Meanwhile, devices using ZnO showed moderate stability, gradually degrading to 0% of their original PCE over 90 h [70,71]. To confirm the reproducibility of PSCs modified by fullerene ETLs, we fabricated 20 PSC devices for each condition, as shown in Figure 2e and the average PCEs of 8.23 ± 0.67% for (ZnON), 10.94 ± 0.38% (ZnO), 13.32 ± 0.35% (IZO), 16.05 ± 0.29% (WIZO) and 8.98 ± 0.59% (w/o ETL) were achieved with the metal oxides and w/o ETL, respectively. To further examine the film structures, their relative surface energies were characterized via water contact angle (θ) (see Figures S6 and S7). ITO surfaces exhibited the lowest contact angles (83°), while ZnON and ZnO films had similar hydrophilic properties of with contact angles of 88° and 86° respectively. Water contact angles of IZO and WIZO films revealed relatively hydrophobic properties with angles of 100° and 101° respectively [67,72].

AFM measurements show that the metal oxide films have a smooth and compact morphology (Figure S3), with root mean squared (RMS) roughness values of 3.05 nm or less. For the interfacial modification of the top surface of the metal oxide layer, PC_61_BM was spin-coated onto the metal oxide film and then annealed at 80 °C for 5 min. Because the fullerene derivative of PC_61_BM is an excellent electron acceptor with a favorable electronic structure with respect to the perovskite absorber, it is expected that it can effectively modulate the interfacial properties of metal oxide [45]. To understand the structure of PC_61_BM films deposited on metal oxide substrate, the surface morphologies were characterized by AFM (Figure 3) and SEM (Figure S4a–h). After the metal oxide layer was coated with a PC_61_BM layer, the surfaces morphologies exhibited only slight differences. The measured RMS values were 0.45 nm for substrate with ITO/PC_61_BM, 0.59 nm for with ITO/ZnON/PC_61_BM, 0.49 nm for substrate with ITO/ZnO/PC_61_BM, 0.42 nm for substrate with ITO/IZO/PC_61_BM, and 0.35 nm for substrate with ITO/WIZO/PC_61_BM. AFM shows that after depositing the PC_61_BM layer, the surface of the substrates is decreased relative to ITO. Therefore, we conclude that by combining the water contact angle and surface roughness by AFM indicate that WIZO is best surface for the spin coating of the perovskite.

Perovskite layers in our devices exhibited large grain sizes and compact morphology, which contributed to the favorable device performance in this study. The rough metal oxide layer became smoother when coated by PC_61_BM. To investigate if the grain structure and surface morphologies were affected by the different types of substrates, SEM measurements were further conducted to study the effect of metal oxide on the morphology of CH_3_NH_3_PbI_3_ phase. Figure 4 shows the SEM images of CH_3_NH_3_PbI_3_ films deposited using CB as the anti-solvent, after drying and 10 min annealing at 100 °C. A relatively uniform film with few pinholes was obtained upon depositing CH_3_NH_3_PbI_3_ on PC_61_BM without a metal oxide layer (Figure 4a). Uneven films with a wide size distribution of round hollows were obtained in the case of perovskite films deposited on substrates with ZnON films (Figure 4b). Although the control film (w/o ETL) and ZnO films show a uniform and dense form, the grain size is smaller than that of IZO and WIZO films. These results are consistent with the SEM topography images, as shown in Supplementary Figure S2. It has been reported that impurities introduce nucleation sites and induce a heterogeneous nucleation and crystal growth, thus, defects and impurities may lead to smaller crystallites [60,61]. Larger grain size indicates lower crystal defect density and trap sites existing in the thin films, which is beneficial for efficient charge transport and reduced charge recombination [73]. As shown in Figure 4c–e, perovskite thin films on substrates with ZnO, IZO, and WIZO exhibited uniform and compact morphologies, with the average grain size of films deposited on WIZO being the largest, indicating that relatively high-quality perovskite films are formed on these substrates. As AFM and water contact angles clearly show, WIZO was able to influence the overlying perovskite layer, leading to larger grain sizes. To confirm the perovskite grain sizes, additional analysis of XRD data was performed using the Scherrer equation, as shown in Figure S5 and Table S2.

**Figure 5:**
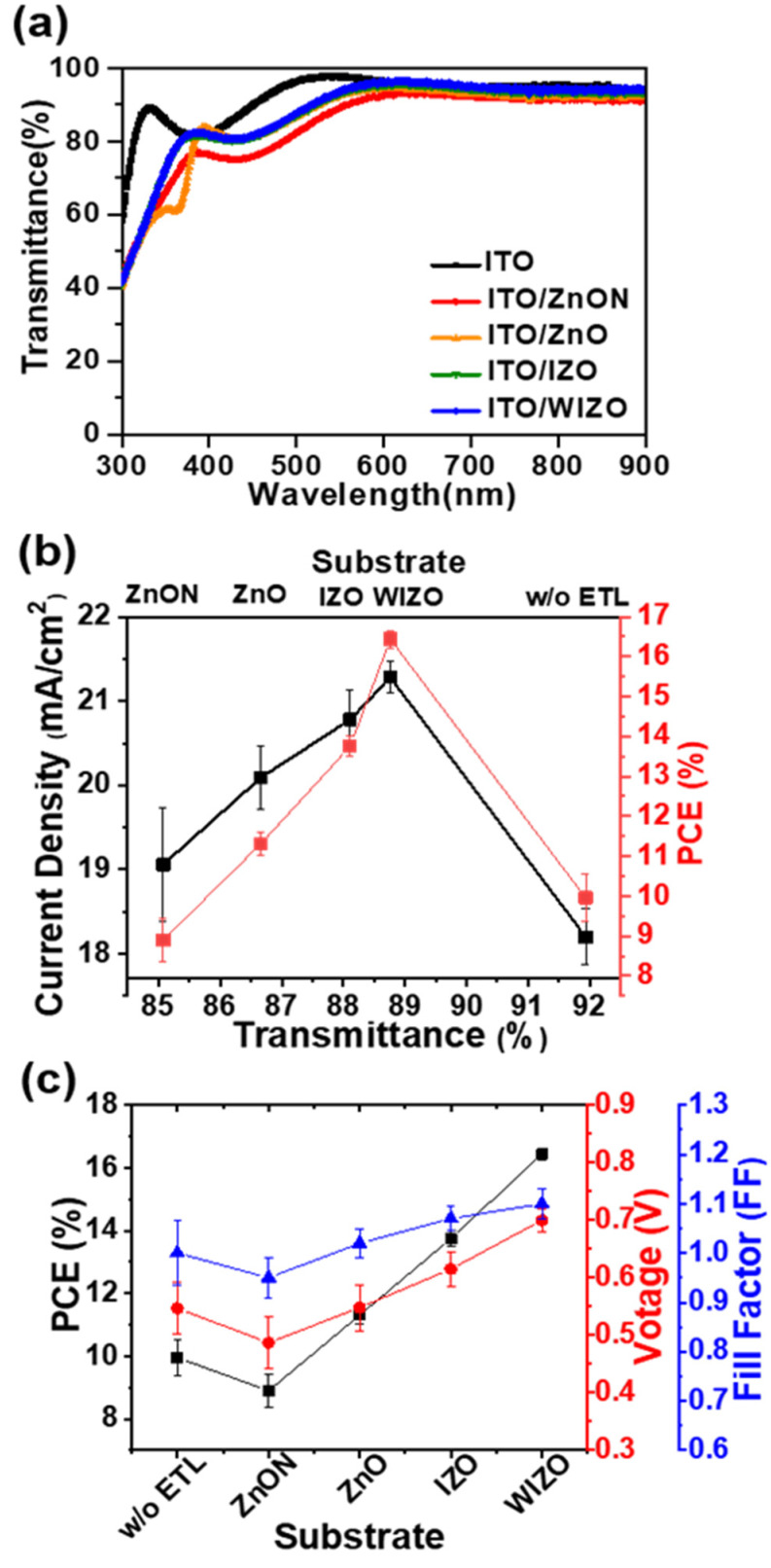
Effect of optical properties on solar cell performance. (**a**) Optical transmittance of metal oxide films deposited on ITO substrates. (**b**) Current density and power conversion efficiency versus transmittance, and (**c**) effect on perovskite PCE, V_OC_, and FF in difference metal oxide substrate of PSCs.

Another possible mechanism by which the oxide films may impact device performance is through their optical properties. Light entering the active layer must first pass through the metal oxide film, thus, light absorbed or reflected by the oxide layer may decrease the amount of light reaching the active layer and consequently decrease the photocurrent. Figure 5 shows the J_SC_ and PCE versus transmittance of substrates with different metal oxides. The photovoltaic performance strongly correlated with the transmittance of the substrates. The average transmittances of the metal oxides were ITO (91.9%), ZnON (85.0%), ZnO (86.6%), IZO (88.1%) and WIZO (88.7%) at the range from 300 to 900 nm, the transmittance of the substrate using ZnON was the lowest. This decrease in transmittance closely tracked with a decrease in J_SC_. To fully understand the influence of the oxides’ optical properties on device performance, optical constants (**n** and **κ**) were measured using spectroscopic ellipsometry and used to model the electric field distribution in the devices [74], as shown in Figure S8. Among the four oxides, the ZnON layer had a slightly higher absorption coefficient (**κ**) and absorbed some incident light before it reached the active layer, resulting in slightly lower field intensity for all layers after ZnON in this device, explaining the low observed J_SC_ in this material. Other than this, the **n** and **κ** values were similar for all four oxide materials, resulting in otherwise similar electric field distributions through the devices. The high transmittance of our composite electrodes explains the high PCE compared to the reference ITO-based solar cells, since more light is transmitted to the absorbing layer, more charge carriers are generated and extracted [52]. This data reveals that doping is an effective strategy to improve the transmittance of ZnO-based electrodes.

## 4. Conclusions

We have introduced an effective way to improve the performance of N–I–P PSCs with a new series of composite metal oxides as electron-transport layers. These composite electrodes give rise to enhanced injection and extraction of photogenerated electrons, avoiding the accumulation of electrons at the ETL/perovskite interface, which in turn leads to a remarkable increase in J_SC_, V_OC_ and FF, and stability of PSCs. Depending on the metal oxides used, the quality of the perovskite film was also affected. The perovskite grain size correlated with the shape of the underlying film, and when without metal oxide was used, a Schottky barrier was formed between ITO and PC_61_BM, so that Ohmic contact was induced using a layer of metal oxide, which is an electron transfer layer. Therefore, it was found that the metal oxide films were found to affect charge transport and charge recombination in the active layer as well. Among the metal oxides investigated in this study, the WIZO metal oxide ETL composition was found to produce optimal results, yielding highly efficient planar PSCs with PCEs of up to 16.44%. The WIZO devices were observed to have the greatest stability and highest PCE of the four new metal oxides. This study demonstrates that sputtered composite metal oxides constitute a functional interface material that can replace existing ETLs, offering significant degree of control over photovoltaic performance and device stability.

## Figures and Tables

**Table 1:** Device parameters of the PVSCs based on metal oxide layer.

Substrates	V_OC_ (V)	J_SC_ (mA cm^-2^)	FF	R_S_ (Ω cm^2^)	R_SH_ (Ω cm^2^)	PCE (%)	
						Champion	Average
No ETL	1.00 ± 0.13	18.20 ± 0.33	0.547 ± 0.09	14.4	173	9.955	8.98 ± 0.59
ZnON	0.95 ± 0.08	19.06 ± 0.53	0.487 ± 0.09	18.4	244	8.90	8.23 ± 0.67
ZnO	1.02 ± 0.06	20.09 ± 0.29	0.548 ± 0.08	15.8	354	11.31	10.94 ± 0.38
IZO	1.07 ± 0.05	20.78 ± 0.25	0.615 ± 0.06	12.4	383	13.75	13.32 ± 0.35
WIZO	1.10 ± 0.06	21.28 ± 0.19	0.700 ± 0.04	7.89	602	16.44	16.05 ± 0.29

## Supplementary Materials

The following are available online at https://www.mdpi.com/2073-4360/12/4/737/s1, Figure S1. UPS spectra of (a) the secondary edge region and (b) the valence band region of metal oxide films on ITO substrate, Figure S2. Grain size distribution of the perovskite thin films produced with different metal oxides, Table S1. Grain size distribution of perovskite thin films with different metal oxides, Figure S3. Topographic images (size: 5 μm × 5 μm) of (a) ITO, (b) ITO/ZnON (c) ITO/ZnO, (d) ITO/IZO, and (e) ITO/WIZO films, Figure S4. Top view SEM images of Top view SEM images of (a) ITO/ZnON thin film, (b) ZnON-sputtered PC_61_BM layer on ITO substrate, (c) ITO/ZnO, (d) ZnO-sputtered PC_61_BM layer on ITO substrate, (e) ITO/IZO, (f) IZO-sputtered PC_61_BM layer on ITO substrate, (g) ITO/WIZO, and (h) WIZO-sputtered PCBM layer on ITO substrate. Insets are magnified images for each film and scale bar is 50 nm, Figure S5. (a) XRD patterns of CH_3_NH_3_PbI_3_ films on different metal oxide/PC_61_BM substrates. The intensities were normalized with respect to the (110) lattice plane. (b) The zoomed in XRD patterns between 13 and 15 degrees for the MAPbI_3_ respectively, Table S2. Measured parameters of CH_3_NH_3_PbI_3_ solar cells, Figure S6. Photos of water droplets on the surfaces of (a) ITO, (b) ITO/ZnON, (c) ITO/ZnO, (d) ITO/IZO, and (e) ITO/W-IZO films deposited on the ITO substrates, Figure S7. Water contact angle images of (a) PC_61_BM, (b) ZnON/PC_61_BM, (c) ZnO/PC_61_BM, (d) IZO/PC_61_BM, and (e) WIZO/PC_61_BM on the ITO substrate, and Figure S8. Electric field distributions in devices using ZnO, (b) ZnON, (c) IZO, and (d) WIZO ETLs.
