# Supplementary material for: Tungsten-Doped Zinc Oxide and Indium–Zinc Oxide Films as High-Performance Electron-Transport Layers in N–I–P Perovskite Solar Cells
Source: Polymers (Basel). 2020 Mar 26;12(4):737. doi: 10.3390/polym12040737 (PMC7240459; doi:10.3390/polym12040737)
Supplement: Supplementary file 1 [file polymers-12-00737-s001.pdf]

Supplementary information for:

Tungsten doped zinc oxide and indium-zinc oxide films as high-performance electron transport layers in N-I-P perovskite solar cells

Ju Hwan Kang,[1,†] Aeran Song,[2,†] Yu Jung Park,[1] Jung Hwa Seo,[1,*] Bright Walker,[3,*] and Kwun Bum Chung[2,*]

[1]*Department of Materials Physics, Dong-A University, 49315, Republic of Korea*

[2]*Division of Physics and Semiconductor Science, Dongguk University, Seoul 04620, Republic of Korea*

[3]*Department of Chemistry, Kyung Hee University, 02453, Seoul, Republic of Korea*

1. **Ultraviolet photoelectron spectroscopy (UPS) Spectra of metal oxide**

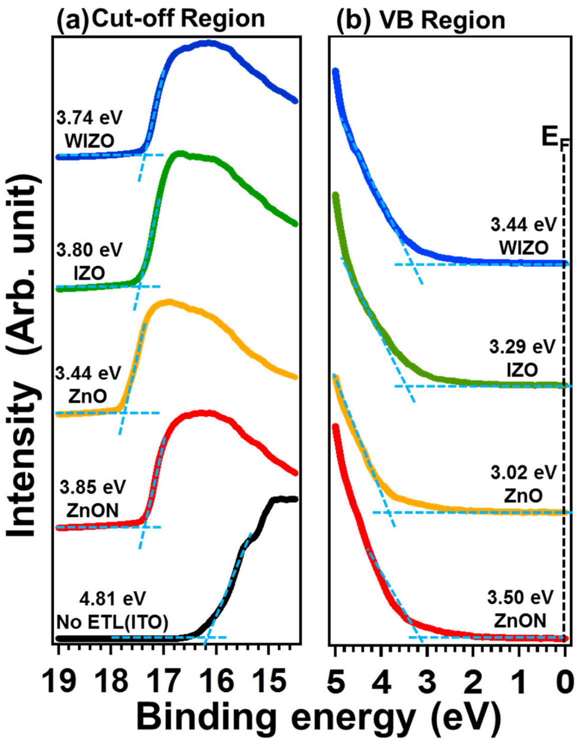

Figure S1. UPS spectra of (a) the secondary edge region and (b) the valence band region of metal oxide films on ITO substrate.

2. **Grain size distribution of the perovskite thin films produced at metal oxide subst rate.**

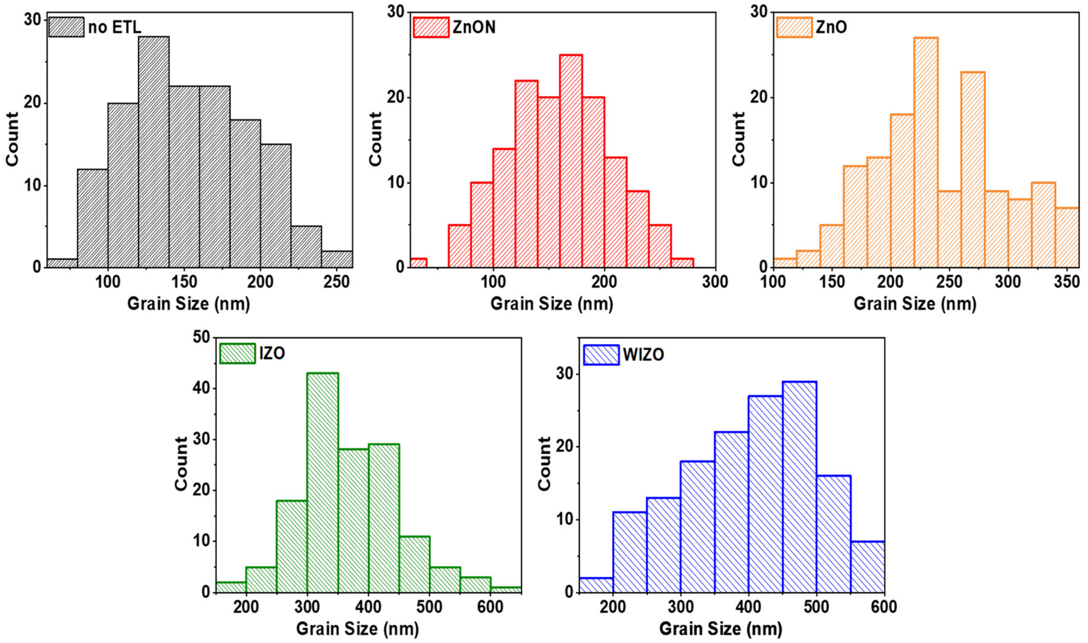

Figure S2. Grain size distribution of the perovskite thin films produced with different metal oxides.

Table S1. Grain size distribution of perovskite thin films with different metal oxides.

Substrate	Size distribution (nm)	Average grain size (nm)
w/o ETL	68 - 252	153
ZnON	65 - 280	157
ZnO	134 - 350	246
IZO	186 - 546	366
WIZO	188 - 594	400

3. AFM images of metal oxide films

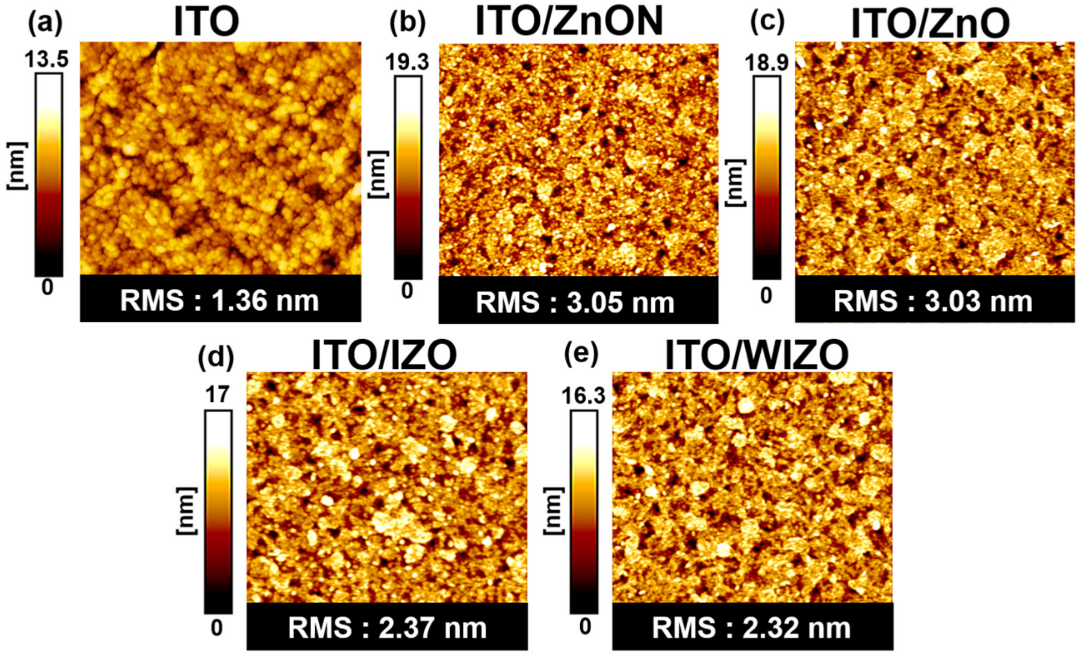

Figure S3. Topographic images (size: 5 μm × 5 μm) of (a) ITO, (b) ITO/ZnON (c) ITO/ZnO, (d) ITO/IZO, and (e) ITO/WIZO films.

4. SEM images of metal oxide films

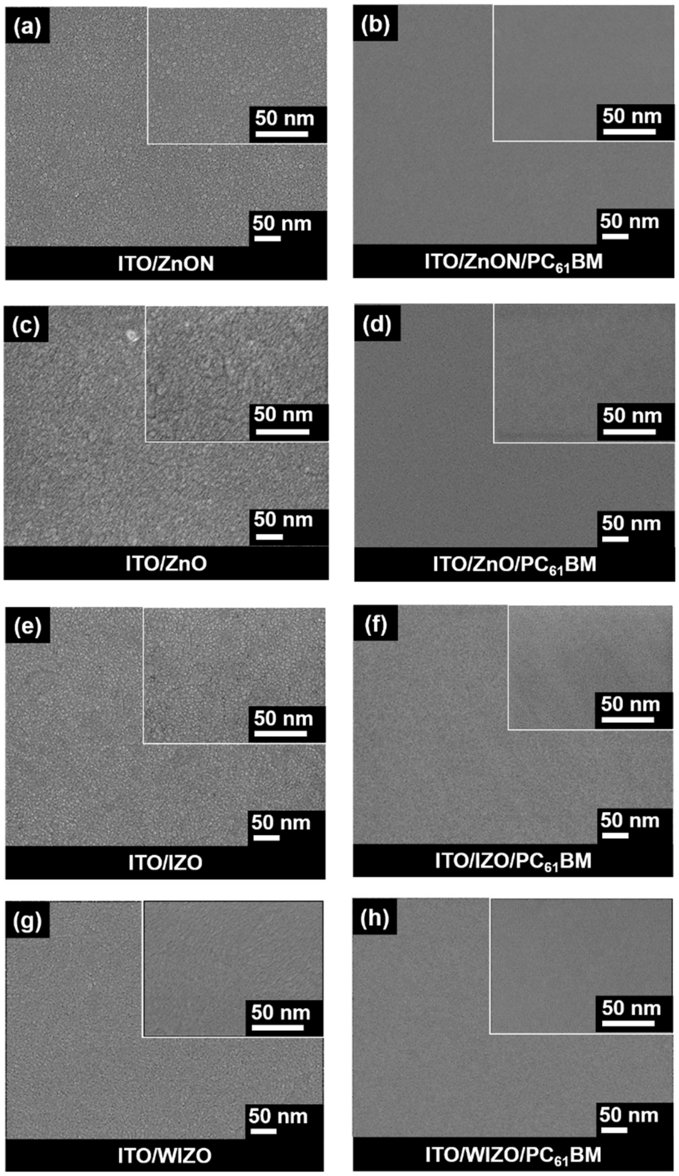

Figure S4. Top view SEM images of Top view SEM images of (a) ITO/ZnON thin film, (b) ZnON-sputtered $PC_{61}BM$ layer on ITO substrate, (c) ITO/ZnO, (d) ZnO-sputtered $PC_{61}BM$ layer on ITO substrate, (e) ITO/IZO, (f) IZO-sputtered PCBM layer on ITO substrate, (g) ITO/WIZO, and (h) WIZO-sputtered $PC_{61}BM$ layer on ITO substrate. Insets are magnified images for each film and scale bar is 50 nm.

5. XRD patterns of $CH_3NH_3PbI_3$ films on different metal oxide

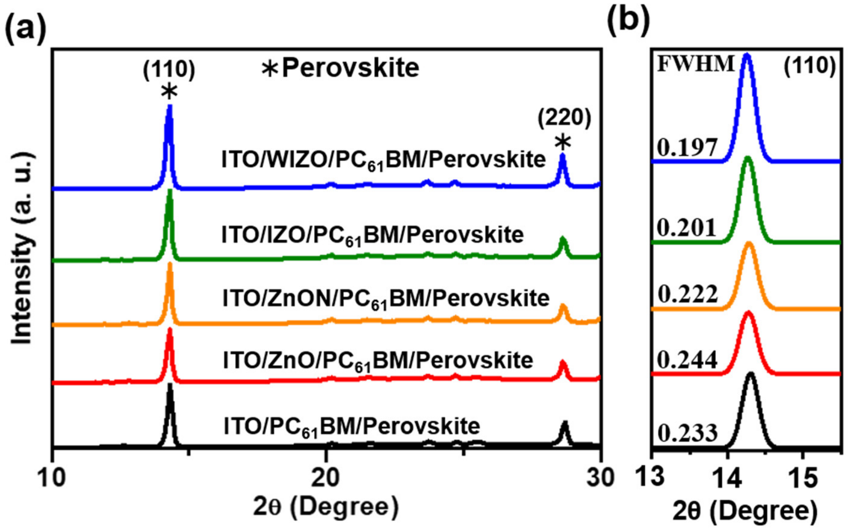

Figure S5. (a) XRD patterns of $CH_3NH_3PbI_3$ films on different metal oxide/$PC_{61}BM$ substrates. The intensities were normalized with respect to the (110) lattice plane. (b) The zoomed in XRD patterns between 13 and 15 degrees for the $CH_3NH_3PbI_3$ respectively.

The corresponding XRD patterns for 2 θ from 10° to 30° are presented in Fig S5 (a). The crystallinity of $CH_3NH_3PbI_3$ films on metal oxide/$PC_{61}BM$ obtained via CB anti-solvent washing was surveyed, by means, of XRD measurements. The diffraction peaks are assigned to the lattice planes (110), and (220) respectively. To understand the metal oxide/$PC_{61}BM$ dependent crystallinity of $CH_3NH_3PbI_3$ films, we took a closer look at the (110) plane illustrated in Fig. S5 (b) to check the FWHM which has direct effect of the crystallinity. It is quite evident that, the FWHM value is went down in order of ZnON > ITO > ZnO > IZO > WIZO. So, by using the value of FWHM to calculate the crystallite size, by using Scherrer equation, and the lattice strain in the film were also calculated and the results are shown in Table S2. The mean crystallite sizes were estimated to be 59.84 nm for ITO, 57.06 nm for ITO/ZnON, 62.80 nm for ITO/ZnO, 69.19 nm for ITO/IZO, and 70.58 nm for WIZO. The improvement of the crystal quality can be deduced from the increase of the diffraction intensity,

in the order ZnON < ITO < ZnO < IZO < WIZO.[1] The 2θ peaks of $CH_3NH_3PbI_3$ films with ITO, ZnON, ZnO, IZO and WIZO as metal oxide are centered at 14.30°, 14.28°, 14.27°, 14.26° and 14.24°, respectively. The average crystalline size can be estimated using the Scherrer equation:

$$k\lambda = \beta D \cos\theta \quad (1)$$

where D is the crystallite size, λ is the wavelength of the radiation (1.54056 Å for CuK_α radiation), k is a constant equal to 0.94, β is the FWHM, and θ is the peak position.

Table S2. Measured parameters of $CH_3NH_3PbI_3$ solar cells.

**Substrate**	**2θ (degree)**	**FWHM**	**d-spacing (nm)**	**Crystallite Size (nm)**
w/o ETL	14.3	0.233	6.18	59.84
ZnON	14.3	0.244	6.18	57.06
ZnO	14.3	0.222	6.18	62.80
IZO	14.3	0.201	6.18	69.19
WIZO	14.2	0.197	6.21	70.58

6. Water contact measurements of metal oxide films

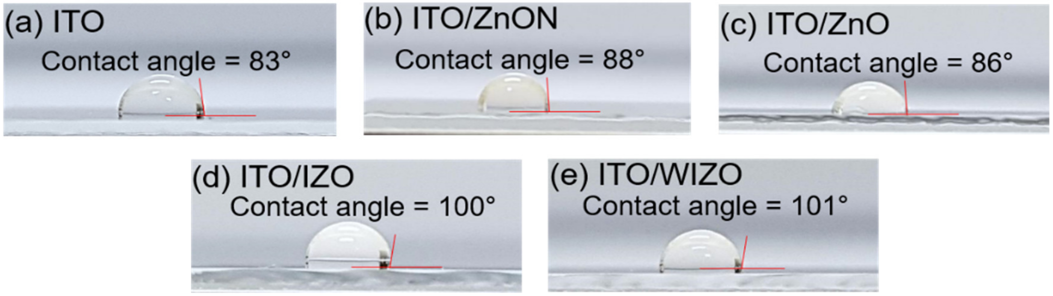

Figure S6. Photos of water droplets on the surfaces of (a) w/o metal oxide, (b) ZnON, (c) ZnO, (d) IZO and (e) WIZO on the ITO substrate.

7. Water contact measurements of metal oxide/PC$_{61}$BM films

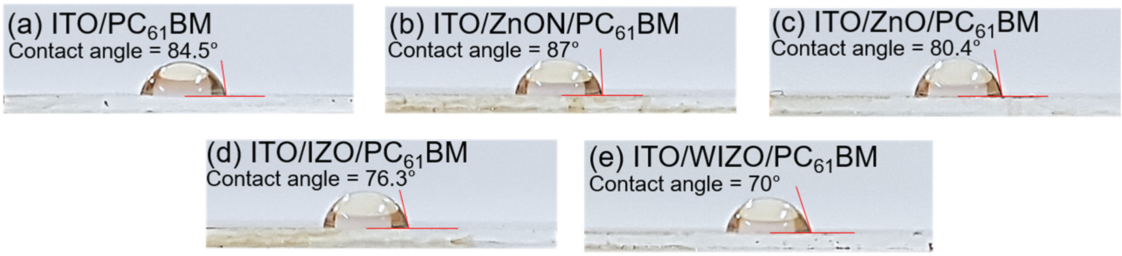

Figure S7. Water contact angle images of (a) PC$_{61}$BM, (b) ZnON/PC$_{61}$BM, (c) ZnO/PC$_{61}$BM, (d) IZO/PC$_{61}$BM, and (e) WIZO/PC$_{61}$BM on the ITO substrate.

8. Electric field distribution in devices

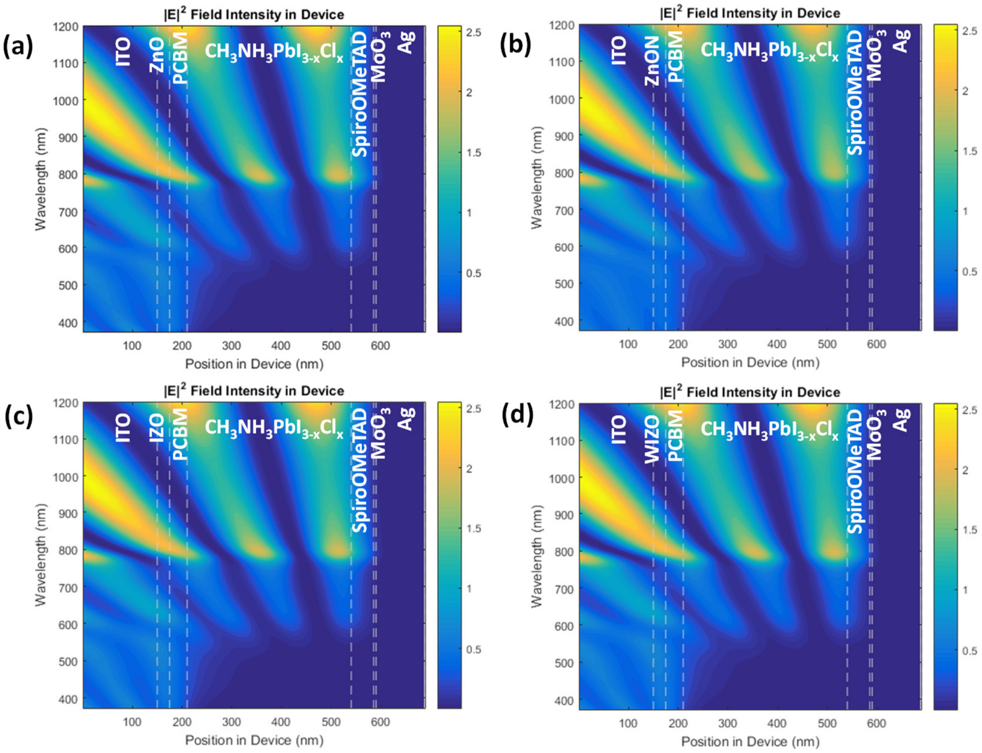

Figure S8. Electric field distribution in devices using (a) ZnO, (b) ZnON, (c) IZO and (d) WIZO ETLs.
